# Plasmid-mediated *mcr-1* colistin resistance in *Escherichia coli* and *Klebsiella spp.* clinical isolates from the Western Cape region of South Africa

**DOI:** 10.1186/s13756-017-0234-8

**Published:** 2017-08-03

**Authors:** Mae Newton-Foot, Yolandi Snyman, Motlatji Reratilwe Bonnie Maloba, Andrew Christopher Whitelaw

**Affiliations:** 1 Division of Medical Microbiology, Faculty of Medicine and Health Sciences, Stellenbosch University, Tygerberg Cape Town, South Africa; 2 National Health Laboratory Service, Tygerberg Hospital, Cape Town, South Africa

**Keywords:** Colistin resistance, *mcr-1*, plasmid-mediated resistance, *E. coli*, *Klebsiella* spp, South Africa

## Abstract

**Background:**

Colistin is a last resort antibiotic for the treatment of carbapenem-resistant Gram negative infections. Until recently, mechanisms of colistin resistance were limited to chromosomal mutations which confer a high fitness cost and cannot be transferred between organisms. However, a novel plasmid-mediated colistin resistance mechanism, encoded by the *mcr-1* gene, has been identified, and has since been detected worldwide. The *mcr-1* colistin resistance mechanism is a major threat due to its lack of fitness cost and ability to be transferred between strains and species. Surveillance of colistin resistance mechanisms is critical to monitor the development and spread of resistance.This study aimed to determine the prevalence of the plasmid-mediated colistin resistance gene, *mcr-1,* in colistin-resistant *E. coli* and *Klebsiella* spp*.* isolates in the Western Cape of South Africa; and whether colistin resistance is spread through clonal expansion or by acquisition of resistance by diverse strains.

**Methods:**

Colistin resistant *E. coli* and *Klebsiella* spp. isolates were collected from the NHLS microbiology laboratory at Tygerberg Hospital. Species identification and antibiotic susceptibility testing was done using the API® 20 E system and the Vitek® 2 Advanced Expert System™. PCR was used to detect the plasmid-mediated *mcr-1* colistin resistance gene and REP-PCR was used for strain typing of the isolates.

**Results:**

Nineteen colistin resistant isolates, including 12 *E. coli*, six *K. pneumoniae* and one *K. oxytoca* isolate, were detected over 7 months from eight different hospitals in the Western Cape region. The *mcr-1* gene was detected in 83% of isolates which were shown to be predominantly unrelated strains.

**Conclusions:**

The plasmid-mediated *mcr-1* colistin resistance gene is responsible for the majority of colistin resistance in clinical isolates of *E. coli* and *Klebsiella* spp. from the Western Cape of South Africa. Colistin resistance is not clonally disseminated; the *mcr-1* gene has been acquired by several unrelated strains of *E. coli* and *K. pneumoniae.* Acquisition of *mcr-1* by cephalosporin- and carbapenem-resistant Gram negative bacteria may result in untreatable infections and increased mortality. Measures need to be implemented to control the use of colistin in health care facilities and in agriculture to retain its antimicrobial efficacy.

## Background

The global increase in antibiotic resistance is extremely concerning as it compromises patient outcome and increases the financial burden on health-care systems [1, 2]. Amongst Gram-negative bacteria, including the *Enterobacteriaceae*, the situation is particularly alarming as the available treatment options for multi-resistant organisms are limited, and there is a paucity of new drugs being developed. The use of β-lactam antibiotics to treat *Enterobacteriaceae* has been severely compromised by the spread of extended-spectrum β-lactamases (ESBLs), which confer resistance to third and fourth generation cephalosporins, resulting in increased carbapenem use. The emergence and spread of carbapenem resistance, primarily mediated by the plasmid-encoded carbapenemases, is therefore of extreme concern [3, 4].

The polymyxins, colistin and polymyxin B, are the “last resort” antibiotics for treatment of infections with carbapenemase producing *Enterobacteriaceae* and in 2012 colistin was reclassified by the WHO as critically important for human medicine [5]. Colistin is a polycationic molecule which interacts with the bacterial outer membrane by displacing divalent cations from the negatively-charged phosphate groups of the Lipid A of the lipopolysaccharide membrane, resulting in cell lysis. Traditionally, colistin resistance was considered to be due to rare chromosomal mutations in the genes encoding the PmrA/PmrB and PhoP/PhoQ two component signalling systems or the negative regulator MgrB [6]. These mutations result in modifications to the Lipid A molecule, or rarely, the complete loss of Lipid A. These chromosomal mutations confer a fitness cost to the organism and are unlikely to be maintained in the absence of colistin selection; and are not transferable to other organisms. In November 2015, the emergence of a novel plasmid-mediated colistin resistance mechanism was described [7]. This colistin resistance is conferred by the *mcr-1* gene which was identified on an IncI2 plasmid, pHNSHP45, isolated from an *Escherichia coli* isolate from a pig in China. The *mcr-1* gene encodes a phosphoethanolamine transferase enzyme which transfers a phosphoethanolamine to Lipid A; conferring resistance to colistin. The plasmid was shown to be transferable by conjugation and transformation, and is stably maintained in *E. coli*, *Pseudomonas aeruginosa* and *Klebsiella pneumoniae* for at least 14 days, in the presence or absence of colistin [7]. Subsequent studies have identified the *mcr-1* gene in various *Enterobacteriaceae*, including *E. coli, K. pneumoniae* and *Salmonella* spp., in Asia, Europe, North America and Africa [8–16].

Plasmid-mediated colistin resistance mechanisms offer no fitness cost and are stably maintained in the absence of colistin selection [7]. These mechanisms can be transferred between bacterial strains and therefore pose a massive risk to the treatment of Gram-negative infections. Distribution of these plasmids amongst carbapenem resistant organisms, especially in the hospital setting, may catalyse a return of the “pre-antibiotic era” for the treatment of infections with Gram-negative bacterial pathogens. This study aimed to determine the prevalence of the plasmid-mediated colistin resistance gene, *mcr-1,* in colistin-resistant *E. coli* and *Klebsiella* spp*.* isolates in the Western Cape of South Africa; and to determine whether colistin resistance is spread through clonal expansion or by acquisition of resistance by diverse strains. Surveillance of colistin resistance mechanisms present in a population is vital for advising effective treatment of bacterial infections and for monitoring the development and spread of resistance.

## Methods

Consecutive colistin resistant *E. coli* and *Klebsiella* spp. isolates were collected from routinely collected clinical specimens processed at the National Health Laboratory Service (NHLS) laboratory at Tygerberg Hospital, as part of convenience sampling, between January and August 2016. The NHLS Microbiology laboratory at Tygerberg Academic Hospital receives specimens from Tygerberg Hospital as well as a number of regional and district hospitals. The hospital serves a drainage area of approximately half of Cape Town (predominantly the Northern and Eastern sub-districts), as well as the West Coast, Cape Winelands and Overberg rural districts. The hospital acts as a referral centre for 4 regional hospitals, 17 district hospitals and over 120 primary health care clinics. The population served is approximately 2.6 million, representing just under half the population of the Western Cape. Microbial identification was done using the API® 20 E system (Analytical Profile Index 20 Enterobacteria) (bioMérieux) or the Vitek® 2 Advanced Expert System™ (bioMérieux) and antimicrobial susceptibilities were determined using the Vitek® 2 Advanced Expert System™. All routinely identified colistin-resistant *E. coli* and *Klebsiella* spp. isolates were collected for the study. Limited specimen information, including specimen type, date and hospital of collection was identified based on the laboratory specimen number. These isolates were not included in a previous study which identified *mcr-1* in South Africa [16]. Colistin minimum inhibitory concentrations (MICs) were determined by gradient diffusion using colistin Etest® strips (bioMérieux). Colistin susceptibility was interpreted using the European Committee on Antimicrobial Susceptibility Testing (EUCAST) Clinical Breakpoints (version 6.0) which defines resistance to colistin in Enterobacteriaceae as MIC >2 μg/mL [17].

PCR detection of the *mcr*-*1* gene was done as previously described, using the primers CLR5 F: 5′-CGGT CAGTCCGTTTGTTC-3′ and CLR5 R: 5′-CTTGGTCGGTCTGTAGGG-3′ [7]. An *rpoB* internal amplification control using RpoB-F 5′-AACCAGTTCCGCGTTGGCCTGG-3′ and RpoB-R 5′-CCTGAACAACACGCTCGGA-3′ was included in the *mcr-1* PCR [18]. PCRs were done using the KAPA Taq ReadyMix PCR Kit (Kapa Biosystems) with 0.4 μM of each primer in a 25 μL reaction volume, using an annealing temperature of 60 °C and 35 cycles. Amplicons were separated on a 2% *w*/*v* agarose gel and detected using the Alliance 2.7 imaging system (UVITec). Sanger sequencing was done to confirm the *mcr-1* amplicons.

Strain typing was done by REP-PCR using primers REP2I: 5′-ICGICTTATCIGGCCTAC-3′ and REP1R: 5′- IIIICGICGICATCIGGC-3′ [19]. *E. coli* strain ATCC 25922 and *K. pneumoniae* strain ATCC 700603 were used as controls for strain typing. PCR was done essentially as previously described using an annealing temperature of 40 °C for 1 min and extension for 8 min at 65 °C, for 30 cycles. Digitised REP-PCR gel images were analysed using GelCompar II version 7.5 (Applied Maths). Banding patterns were normalised to the KAPA™ Universal Ladder (Kapa Biosystems) and band intensity was not evaluated. Similarity between the profiles was calculated with the band matching Dice coefficient and dendrograms for each species were produced by the unweighted pair group method with arithmetic averages (UPGMA) and neighbour-joining algorithms. Identical strains were defined as isolates with >97% similarity, closely related isolates with ≥95% similarity; isolates with <95% similarity were defined as unrelated strains, based on the UPGMA dendograms [20].

## Results

Twenty-one colistin-resistant isolates were collected over the 7 month period between January and August 2016, based on Vitek® 2 susceptibility testing (*n* = 14 *E. coli*, *n* = 6 *K. pneumoniae*, *n* = 1 *Klebsiella oxytoca*) (Table 1). These isolates were identified from specimens collected from 19 patients from eight hospitals in the Western Cape region; Hospital A (*n* = 6), Hospital B (*n* = 3), Hospital C (*n* = 3), Hospital D (*n* = 2), Hospital E (*n* = 2), Hospital F (*n* = 1), Hospital G (*n* = 1) and Hospital H (*n* = 1) (Table 1). The majority of isolates were obtained from urine specimens. Two *E. coli* (CEC12 and CEC15) and two *K. pneumoniae* (CK1 and CK7) isolates were each obtained from urine specimens from the same patient taken at least 2 months apart. All of the *E. coli* isolates, with the exception of CEC10, are susceptible to 3rd and 4th generation cephalosporins, while four of the six *K. pneumoniae* isolates are resistant to both, one of which is also resistant to carbapenems (CK2).

**Table 1:** Specimen details, colistin susceptibilities and presence of *mcr-1* in colistin resistant isolates

Species	Isolate	Specimen type	Date of collection	Hospital	Vitek MIC (μg/ml)	Etest MIC (μg/ml)	*mcr-1* PCR	Additional antibiotic resistance
*E. coli*	CEC1	Urine	25/01/2016	A	4 (R)	4 (R)	+	SXT,
	CEC2	Urine	16/01/2016	F	4 (R)	4 (R)	+	SXT, CIP, CXM(I)
	CEC3	Urine	15/01/2016	D	16 (R)	4 (R)	+	none
	CEC4	Urine	16/01/2016	E	8 (R)	4 (R)	+	SXT, AMP, CXM(I)
	CEC5	Urine	01/02/2016	H	16 (R)^c^	0.5 (S)	-	AMI(I)
	CEC7	Superficial abdominal swab	27/01/2016	D	8 (R)	4 (R)	+	SXT, AMP, AMC(I), CIP, CXM(I), TZP(I)
	CEC8	Urine	12/02/2016	A	8 (R)	4 (R)	+	SXT, AMP, CIP
	CEC9	Urine	16/02/2016	B	4 (R)	2 (S)	+	SXT, AMP, CIP
	CEC10	Urine	16/02/2016	C	4 (R)	2 (S)	+	AMP, AMC(I), CIP, CXM, CTX, CAZ, FEP, AMI(I), TZP(I)
	CEC11	Urine	03/03/2016	B	8 (R)	4 (R)	+	SXT, AMP,
	CEC12a	Urine	23/05/2016	A	4 (R)	4 (R)	-	SXT, AMP, AMC(I)
	CEC13	Urine	10/06/2016	G	4 (R)	4 (R)	+	SXT, AMP, CIP, CXM(I), FOX(I)
	CEC14	Urine	27/07/2016	A	4 (R)^c^	0.5 (S)	-	none
	CEC15^a^	Urine	23/07/2016	A	4 (R)	4 (R)	-	SXT, AMP
*K. pneumoniae*	CK1^b^	Urine	20/05/2016	E	4 (R)	2 (S)	+	AMP, AMC, CIP, CXM, CTX, CAZ, FEP, TZP(I)
	CK2	Sputum	17/06/2016	A	16 (R)	4 (R)	+	SXT, AMP, AMC, CIP, CXM, FOX, CTX, CAZ, FEP, GEN, AMI, TZP, ETP, IPM, MEM
	CK5	Urine	17/07/2016	C	4 (R)	0.5 (S)	+	AMP, CXM
	CK6	Urine	26/07/2016	A	16 (R)	Isolate lost during culture		SXT, AMP, AMC, CIP, CXM, FOX, CTX, CAZ, FEP, GEN, AMI(I), TZP
	CK7^b^	Urine	12/08/2016	E	16 (R)	4 (R)	-	SXT, AMP, AMC, CIP, CXM, CTX, CAZ, FEP, GEN, TZP(I)
	CK8	Sputum	02/07/2016	C	4 (R)	0.5 (S)	+	AMP, GEN(I), AMI(I)
*K. oxytoca*	CK3	Superficial skin swab	21/06/2016	B	16 (R)	0.25 (S)	+	AMP

^a^Successive *E. coli* isolates obtained from the same patient. ^b^Successive *K. pneumonia* isolates obtained from the same patient. ^c^Repeat Vitek susceptibility testing redefined the colistin MIC as 0.5 μg/mL; both these isolates were excluded from further analysis. *R* resistant, *I* intermediate, *S* susceptible, *SXT* trimethoprim-sulfamethoxazole, *CIP* ciprofloxacin, *CXM* cefuroxime, *AMP* ampicillin, *AMI* amikacin, *AMC* amoxicillin-clavulanic acid, *TZP* piperacillin-tazobactam, *CTX* cefotaxime/ceftriaxone, *CAZ* ceftazidime, *FEP* cefepime, *GEN* gentamicin, *ETP* ertapenem. *IPM* imipenem, *MEM* meropenem

There was good correlation between the Vitek and Etest colistin susceptibility results in the *E. coli* isolates. For 12 of the 14 *E. coli* isolates the MICs agreed, or showed a single fold dilution difference (Table 1). The remaining two *E. coli* isolates (CEC5 and CEC14) were found to be colistin susceptible by Etest (MIC = 0.5 μg/mL). The VITEK susceptibilities were repeated on these isolates; and repeat testing found the colistin MIC of both isolates to be 0.5 μg/mL; these isolates were reclassified as colistin susceptible and excluded from the analysis. One *K. pneumoniae* isolate, CK6, was lost during subsequent culture and was excluded from further analysis. The correlation between the Vitek and Etest susceptibility results was poor for the *Klebsiella* spp. isolates; with all but one isolate showing a greater than 1 fold dilution difference between the two testing methods. Therefore, we found 19 colistin resistant isolates (12 *E. coli*, 6 *K. pneumonia*, and 1 *K. oxytoca*)*.*

The *mcr-1* gene was detected in 15 out of 18 (83%) confirmed colistin-resistant isolates, including 10/12 *E. coli* isolates and 5/6 *Klebsiella* spp*.* isolates (Table 1). As no *mcr-1* positive control strain was available, selected *mcr-1* amplicons from both *E. coli* and *Klebsiella* spp. isolates were sequenced and shown to share 100% identity with the published *mcr-1* gene sequence (Genbank accession number: NG_050417.1) [7]. Four *mcr-1* positive *Klebsiella* isolates were reported to be colistin susceptible by the Etest method. The two *mcr-1*-negative colistin resistant *E. coli* isolates (CEC12 and CEC15), were isolated from the same patient.

**Fig. 1:**
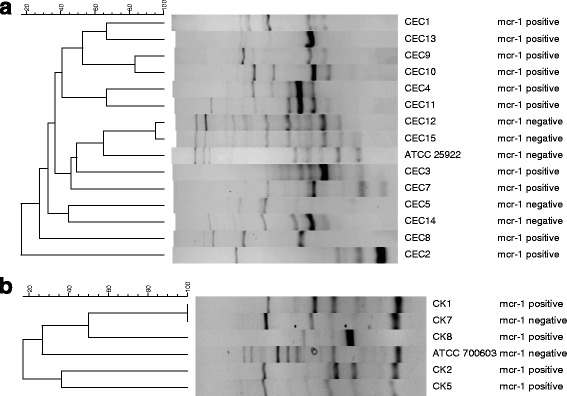
UPGMA dendograms representing the relatedness of **a** *E. coli* and **b** *Klebsiella pneumoniae* strains. Clustering was consistent between the UPGMA and neighbor-joining dendograms (data not shown)

Strain typing using REP-PCR identified 11 unrelated strain types amongst the *E. coli* and 4 amongst the *Klebsiella* spp. isolates (Fig. 1). Two genetically related *E. coli* isolates (CEC12 and CEC15), with 95% similarity, and two identical *K. pneumoniae* isolates (CK1 and CK7) were respectively identified from the same patient. Isolate CK1 was *mcr-1*-positive, while CK7 was *mcr-1*-negative, even after repeating the PCR. Both isolates were however colistin resistant, although CK7 had a higher colistin MIC (16 μg/mL) than CK1 (4 μg/mL).

## Discussion

The plasmid-mediated *mcr-1* colistin resistance gene was found to be the predominant colistin resistance mechanism amongst *E. coli* and *Klebsiella* spp. clinical isolates in the Western Cape of South Africa, present in 83% of colistin resistant isolates. These *mcr-1* positive isolates were obtained from seven hospitals across the Western Cape, indicating that this resistance mechanism is widespread in the province. Previously, *mcr-1* has been reported in eight colistin resistant *E. coli* isolates from patients in Johannesburg and Pretoria, and one from Cape Town [16]; however the presence of *mcr-1* in clinical *Klebsiella* isolates has not been previously described in South Africa. This highlights the need for screening for *mcr-1* in other Gram negative organisms in addition to *E. coli.* The presence of the *mcr-1* colistin resistance mechanism in *K. pneumoniae* is particularly concerning in light of the high prevalence of ESBL-production as well as ongoing emergence of carbapenem resistance amongst *K. pneumoniae* in South Africa [21, 22]. The *mcr-1* gene was detected in two *K. pneumoniae* isolates which are resistant to 3rd and 4th generation cephalosporins, one of which is also resistant to carbapenems, indicating that these highly resistant organisms are already present in our population.

The *mcr-1* gene was detected in diverse strains of *E. coli* and *K. pneumoniae* from geographically diverse hospitals, indicating that this plasmid-mediated colistin resistance mechanism is not distributed clonally, but mediated by multiple independent acquisitions of *mcr-1* containing plasmids. Six of these hospitals are regional or district hospitals, where colistin use is extremely uncommon, and it is probable that these isolates are present in the community, rather than arising as a result of selective pressure in hospitals. This is consistent with previous data from South Africa which showed that the *mcr-1* positive *E. coli* isolates from Johannesburg and Pretoria were unrelated strains, containing *mcr-1* on 3 different plasmid types [23]. Plasmid typing has not yet been done on the isolates in this study, therefore it cannot be concluded whether the dispersion of *mcr-1* in the Western Cape is due to the spread of a single *mcr-1* containing plasmid amongst different strains and species, or whether *mcr-1* is present on multiple plasmid types in this community.

The two genetically identical *K. pneumoniae* isolates, obtained from the same patient, appear to have distinct colistin resistance mechanisms. The first isolate contained the *mcr-1* gene, while the second was *mcr-1*-negative. Further studies are required to explain this finding, which may be due to loss of the *mcr-1* plasmid in combination with development of chromosomal colistin resistance mutations, or acquisition of an alternative plasmid-mediated gene such as *mcr-2* [24]. New mutations in *mcr-1*, resulting in increased colistin MICs and loss of one or both primer binding sites, is another speculative explanation. Both of these isolates are resistant to 3rd and 4th generation cephalosporins and the second isolate had also acquired resistance to trimethoprim-sulfamethoxazole and gentamicin, which may be linked to acquisition of additional plasmids. The mechanism/s of colistin resistance in the other *mcr-1* negative isolates also requires further investigation.

The high prevalence of the *mcr-1* colistin-resistance gene in China was attributed to the widespread use of colistin in their veterinary sector [7]. In South Africa, an increased prevalence of colistin resistance was observed in *E. coli* obtained from chickens in the last quarter of 2015, as part of the MIC surveillance program, and 79% of these colistin resistant *E. coli* isolates (19/24) were *mcr-1* positive [25]. As a result of these findings, as well as the presence of *mcr-1* in human isolates in South Africa, and colistin’s position as an antibiotic of last resort for human health, the South African Veterinary Council (SAVC) recently recommended that colistin not be used in feed producing animals unless its use can be justified by a sensitivity test showing that it is the only therapeutic option available [25]. Prudent use of colistin in agriculture is vital to prevent further spread of the *mcr-1* gene to other bacteria and to retain its use in humans and animals [25, 26].

In July 2016, EUCAST issued a statement recommending that the Etest not be used for colistin MIC determination, after evaluating its use on a collection of isolates with and without known colistin resistance mechanisms [27]. Results indicated that the Etest underestimates MIC values. Furthermore, In the December 2016 issue of the CLSI AST News Update, the Clinical Laboratory Standards Institute (CLSI)/EUCAST Joint Working Group recommended that broth microdilution, without surfactant, be used as the reference method for testing colistin and that disk and agar gradient diffusion methods not be used as they yield unacceptably high error rates [28]. This is consistent with the findings in this study, which found that Etest MICs were typically lower than those of the Vitek, although considerably more so for *Klebsiella* spp. in which the Etest MICs in 5 of the 6 isolates were at least 2 dilutions lower, even in the presence of the *mcr-1* resistance gene.

The isolates included in this study exhibited a range of antibiotic susceptibility profiles. The *E. coli* isolates were all susceptible to carbapenems, and only one isolate was resistant to cephalosporins and six to the fluoroquinolone ciprofloxacin. However, four of the six *K. pneumoniae* isolates were resistant to 3rd and 4th fourth generation cephalosporins, one of which was also resistant to carbapenems. Other studies have also detected the *mcr-1* resistance gene in isolates which harbour plasmid-mediated ESBL and carbapenemase genes [29–32], and notably, *mcr-1* was found to be present on the same plasmid as an ESBL gene in an *E. coli* isolate in France [33]. This highlights the threat of widespread dispersion of this resistance mechanism and its introduction into more resistant strains. New antibiotics are unlikely to solve the antibiotic resistance problem in the near future, and surveillance of colistin resistance and the prudent use of colistin in humans and animals are vital to retain colistin activity.

## Conclusions

The plasmid-encoded *mcr-1* gene is the predominant colistin resistance mechanism in *E. coli* and *Klebsiella* spp. isolates from clinical specimens in the Western Cape of South Africa. The *mcr-1* gene was detected in unrelated strains from patients at various hospitals throughout the province, suggesting that the *mcr-1* gene has been acquired by multiple strains and is not clonally spread. The presence of *mcr-1* in both *E. coli* and *K. pneumoniae* isolates is of concern; the presence of *mcr-1* in isolates resistant to 3rd and 4th generation cephalosporins and carbapenems is alarming, and highlights the threat of potentially untreatable infections. Increased surveillance of colistin resistance mechanisms to monitor their acquisition and spread is vital, and ongoing efforts to ensure the judicious use of colistin (and indeed all antibiotics) both in agriculture and in health-care facilities are welcomed.

## Abbreviations

ESBL: Extended-spectrum β-lactamase
EUCAST: European Committee on Antimicrobial Susceptibility Testing
HREC: Health Research Ethics Committee
MIC: Minimum inhibitory concentration
NHLS: National Health Laboratory Service
SAVC: South African Veterinary Council
UPGMA: Unweighted pair group method with arithmetic averages
